# Australian Women's Fertility Experiences prior to a Termination of Pregnancy

**DOI:** 10.1155/2014/794380

**Published:** 2014-02-20

**Authors:** Wendy Abigail, Sheryl de Lacey

**Affiliations:** School of Nursing & Midwifery, Flinders University, P.O. Box 2100, Adelaide, SA 5001, Australia

## Abstract

*Objective*. This research aimed to investigate the fertility management of women aged over 30 years prior to a termination of pregnancy (TOP) to inform primary health care service delivery providers and policy makers. *Design*. An ethically approved, two-phase sequential explanatory mixed methods design was used. This paper reports on part of that study. *Setting*. The study was conducted in five South Australian TOP clinics. *Patients*. Women aged over 30 years attending for a TOP in 2009 were invited to participate. *Interventions*. The Contraception Sexual Attitude Questionnaire (modified version) of women attending termination of pregnancy services was used. *Main Outcomes Measures*. Quantitative data analysis utilized SPSS V16 where simple descriptive statistics were described. *Results*. There were 101 questionnaire respondents where 70.5% were Australian women, predominantly married and with children. Women used contraception but experienced method failure, were beginning a new method, or were afraid of side effects. Risk-taking behaviours were reported such as putting the possibility of pregnancy out of their mind, getting carried away and not thinking of pregnancy risk, or frequently having unprotected intercourse. *Conclusion*. Service delivery needs to include age specific programs, and policy makers need to include policies which are adequately funded and evaluated. Further research is required to provide greater depth of knowledge in this area.

## 1. Introduction

Women's choice around reproduction is increasingly the subject of community concern. In many Western cultures, population growth has ceased and women's decision making about controlling fertility to delay childbearing is being examined [1].

The trend towards later childbearing is associated with growing concern about fertility rates and population growth. Fertility rates in Australia, and many other countries (such as New Zealand, USA, UK, and Canada), show a downward trend from the 1950s but this trend has increased since the early 2000s [1]. The total fertility rate in Australia in 2007 was 1.93 babies per woman, a rate which is below the population “replacement level” of 2.1 babies per woman [1, 2]. In South Australia in 2010 the total fertility rate (TFR) was 1.87 babies per woman [3]. An Australian Longitudinal Study on Women's Health (ALSWH) found that, in 2006, 60% of women aged between 28 and 33 years had not had children [4]. By comparison 50% of women born between 1946 and 1951 had had their first child before 24 years of age [4].

Many factors impact on women's decisions including economic conditions, greater access to part-time work and more flexible work arrangements, lifestyle choices such as careers, self-development and greater freedom, shifting social attitudes to childlessness, and changing patterns of partnership formation [1, 5]. A recent review which addressed the question of why women postpone childbearing highlighted social issues such as stability of partnerships and ability to find a partner who will participate in reproduction and parenting [6]. the increased availability of contraception and termination of pregnancy (TOP) has undoubtedly contributed significantly to women's control on when they reproduce.

Trends in TOP vary and accurate worldwide prevalence is difficult to establish due to differences in laws and reporting mechanisms between and within countries [7]. However, estimates show that 44 million women around the world terminate pregnancies each year (28 per 1000 reproductive aged women) [8]. In Australia where TOP is generally considered lawful, it is estimated that between 70,000 and 85,000 TOPs are performed each year which, in 2003, equated to 19.7 per 1000 women of reproductive age [9–12].

Since 2007, women aged 40–44 years were the group that showed the greatest increase in fertility rates in Australia [13]. In South Australia, childbearing data shows that more women gave birth in the 30–34 years than in the 25–29 years age group [13]. Additionally, the proportion of women aged over 35 years in South Australia giving birth was 21.1% of all women [13]. However in South Australia, between 1996 and 2006, there was also an increased trend in women aged over 30 years having a TOP [14].

In order to address the issues of delayed childbearing and increased TOP in this reproductive age group, it is important to understand the experience of women in the age group of 30+. Moreover, a previous review showed a lack of research worldwide that includes women in this age group and little detailed information available to inform service provision in Australia [15] and elsewhere. Hence, the objective of this study was to explore the fertility management experiences of women aged 30+ years who had an unwanted pregnancy and were having a TOP. It is aimed that the knowledge gained from this research will provide sound evidence-based information to inform clinicians working in clinical/community settings, as well as contributing to health policies, education, and health promotion activities, and encourage further research in this sensitive area of health care.

## 2. Material and Methods

This study was conducted in South Australia from 2009 to 2011. In 2008, the population of South Australia was approximately 1.6 million persons [16], of which 73.1% resided in the Adelaide statistical division [17]. South Australia is a multicultural society but according to the latest census data available [18], South Australian residents were predominantly born in Australia [19]. In 2008, it was estimated that the female resident population in South Australia aged between 15 years and 44 years was 319,465, with over half of those being women aged 30–44 years [13].

Approval to conduct the study was obtained from Flinders University Clinical Research Ethics Committee, Central Northern Adelaide Health Service Ethics of Human Research Committee, and the Children, Youth and Women's Health Service Human Research Ethics Committee.

The study used a two-phase pragmatic sequential explanatory mixed methods design as described by Ivankova et al. [20]. This paper reports on Part One in Phase One of this project which consisted of a dominant quantitative phase in which women aged over 30 years were recruited from patient groups within five South Australian metropolitan public hospital TOP services. These services collectively conducted 83% of TOPs in South Australia for women aged over 30 years (personal communication Tucker and Sage, Epidemiology Branch, South Australian Government, 12 February 2008). Women, who were younger than 30 years, were not proficient in written or spoken English, and women who were terminating their pregnancy due to foetal abnormality were excluded from the study. All women who attended these services and met the inclusion criteria had equal opportunity to voluntarily participate.

There was no sampling selection process required for this population as all women who attended the five TOP services during the three months of the data collection period were eligible to complete the questionnaire according to the selection criteria. This constituted a census population (as opposed to a survey where only part of the population is selected), as information was attempted to be collected from every woman who attended one of the five TOP services for a TOP who wish to participate [21]. To calculate the possible population number (*N*) for the study, data from each of the five services was examined for 2006, the latest available statistics as of February 2008 [22]. Sample size was assessed using a sample size calculator which required input of the expected population size for the given period (which was based on the 2006 data), a margin of error set at 5%, confidence level set at 95%, and with a response distribution of 50% [23]. The projected sample size for the data to be representative of the women attending the five TOP services, for three months of data collection, was 180 responses.

A questionnaire was developed which comprised two modified instruments, the first of which is reported in this paper: the Contraception and Sexual Attitude Questionnaire (CSAQ) [24]. The modified CSAQ questionnaire had been previously validated; however, to further improve the validity and reliability of the modified questionnaire a focus group of sexual and reproductive health care professionals examined the content for missing or inappropriate questions and suitable wording. The modified questionnaire consisted of six themes (contraception, pregnancy risk-taking, fertility, attitudes, partner/relationship, and doctor issues) with 44 questions from which women could select the statement(s) that described them or did not describe them.

The questionnaire was then pilot tested for a two-week period in one facility to identify any unforeseen issues. Minor editorial changes only were made such as the description of the time involved to complete the survey.

To avoid any potential bias in data collection, in four services women were provided with information and the questionnaire by trained clinic staff prior to their TOP. The fifth service chose to distribute these by mail along with other admission forms prior to the TOP appointment. Women in all services were provided with a prepaid envelope to return the questionnaire or they could submit it via a locked box at the service provider's clinic. In all cases the women remained anonymous to the researcher and their privacy was protected. The initial three-month data collection period was extended due to low response rates and occurred from January to May 2009.

## 3. Results

Statistical analyses were conducted using the Statistical Package for Social Sciences (SPSS, v.16.0). The preliminary analysis began by calculating the frequencies within each of the sociodemographic and obstetric categories as well as the responses to the survey questions. Simple descriptive methods were used to summarize the data collected on the questionnaires allowing for categorization, listing and description of the findings [25].

There were 101 women (mean age: 35 years) who responded to the questionnaire (response rate: 27.4%). The sociodemographics of the study are presented in Table 1 and obstetric history in Table 2. Overall, the majority resided in the metropolitan area, were English speaking Australians', were employed, had completed secondary schooling, were in stable married relationships, were not eligible for a healthcare/pension card, had children already, had no history of miscarriages or ectopic pregnancies, and had not had a termination of pregnancy previously.

To rectify the potential bias due to the low response rate and to improve the representativeness of the sample, data were weighed to the population of the women attending the five service providers during the study period. This involved examining the total population of women who attended the services for the study period by using aggregated data which was provided by the South Australian Government data collection agency (personal communication Tucker, G, 11th November 2009). Simple descriptive statistics were then calculated using the weighted ordinal and nominal data. The data output provided frequencies, percentages of the overall respondents in the study, and valid percentages of only the respondents who recorded responses to the questions.  Table 3 shows the results of women's experiences of fertility management.

### 3.1. Contraception

Over a third of the women were using contraception when they became pregnant but it did not work (*n* = 43, 42.9%). Approximately one in three women were commencing a new contraceptive method (*n* = 31, 31.0%), were afraid of contraceptive side effects (*n* = 32, 32.0%), thought they were in a safe period of their menstrual cycle (*n* = 31, 30.8%), or thought pregnancy could not happen to them (*n* = 30, 29.5%). One in five women had not planned on having intercourse for a while (*n* = 19, 19.3%) or just did not get around to obtaining contraception (*n* = 21, 21.2%).

### 3.2. Risk-Taking

There were a number of responses related to pregnancy risk-taking. Over a third of the women put the possibility of pregnancy out of their mind (*n* = 36, 36.3%). Approximately one in five women got carried away before they thought about contraception (*n* = 21, 21.4%), had had intercourse frequently without using protection (*n* = 21, 21.0%), and were affected by alcohol at the time (*n* = 19, 18.6%). Approximately one in ten women were not concerned about the risk of becoming pregnant (*n* = 12, 11.8%), felt the risk of pregnancy was worth it on that occasion (*n* = 12, 11.5%), and did not think they would get pregnant as they had intercourse so infrequently (*n* = 11, 11.0%).

### 3.3. Fertility

One in five women (*n* = 20, 20.3%) indicated that they did not think they were fertile at the time. This was the only notable response in the fertility theme.

### 3.4. Attitudes

Approximately one in 10 women responded that they knew if they got pregnant they could get an abortion (*n* = 9, 9.0%). This was the only option chosen in the theme of attitudes.

### 3.5. Partner/Relationship

There were 14 options in the partner/relationships theme. 13 women (*n* = 13, 13.2%) indicated that their sexual partner was supposed to withdraw but did not. Responses to the other options in this theme were low and ranged from 0 to 9 (mean 3.77) responses. These other options with low responses were related to contraception issues, such as not being able to agree on which method to use (*n* = 8, 7.8%), the partner not wanting to use contraception (*n* = 7, 7.4%) and the partner would not use contraception (*n* = 6, 5.7%); and other relationship issues such as the partner wanted a baby even though the woman did not (*n* = 9, 8.6%) and the women loved the partner and nothing else mattered (*n* = 7, 6.7%).

### 3.6. Doctor Issues

There were three options in the doctor issues theme with only a low number of responses selected.

## 4. Discussion

This study found that there are many factors experienced by women aged over 30 years in relation to their fertility management prior to a TOP. Of particular note are contraception issues, relationship dynamics, and risk-taking behaviours.

Although women with an unplanned pregnancy are often blamed for failing to take adequate precautions, the most common reported contraceptive issue for women in the study was that they took precautions but the contraceptive method failed. In addition to contraceptive failure women reported experiencing problems related to commencing a new contraceptive, were afraid of side effects, or thought they were in a safe period of their cycle. Additionally, there were other issues concerning contraception nonuse where women made choices not to take/use contraception depending on their situation. Loxton and Lucke [4] reported that a woman's use and choices of contraception change over her lifetime depending on circumstances such as her intention to conceive and availability of new methods. Other studies have shown that contraceptive use is influenced by age and parity, and the stage of life a woman had reached, that is, whether she was at the beginning or at the end of childbearing [26, 27].

In this study, women aged over 30 years reported contraception use was not always required depending on their situation. The majority of women in this study were either not married, in de facto partnerships or not in a relationship, and three quarters of the women already had children. This finding reflects general trends in TOP which show higher rates in women aged over 30 years (particularly in Southern and Eastern Europe) and which may be attributed to contraception effectiveness/efficacy/use/non-use, desire for less children, completed childbearing, and/or delayed relationship formation [28]. Notably, of those in relationships only half perceived their relationship to be stable.

According to Dominguez-Folgueras and Castro-Martin [29], family formation has changed markedly in recent decades because marriage has been replaced by cohabitation. These authors argue that relationship formation impacts on fertility because those who postpone marriage and are in de facto relationships also postpone childbearing [29]. Other studies have found that women delay childbearing due to partnership issues such as multiple partners before childbearing, difficulty in finding a partner, unstable unions, high levels of divorce, women retreating from marriage, and increase in de facto relationships [6, 30]. As some women in this study indicated, contraception was perceived to not be required as they did not usually have or plan to have intercourse. This is likely to be directly related to their relationship status. Further, some women reported taking risks because they believed that they would not get pregnant, evidently because they had had coitus often without using contraception and had not conceived in the past. Kero et al. [31] reported similar responses as being the most common in their study on contraceptive risk taking and TOP. According to Törnbom and Möller [32], this type of risk-taking can lead to a feeling of safety which allows the risk taking to continue. Additionally, women may believe they have low fertility levels [32]. Helström and colleagues linked risk taking to disbelief about fertility levels [33]. This finding was supported in this study where women reported that they did not think they were fertile. A US study reported that older women were at risk of unwanted pregnancy due to taking contraceptive risks, especially if divorced [34]. Kumar et al. found that some women took risks for months or years assuming their fertility was low [35]. Where women were taking/using less effective or no contraception, were single, living alone, childless or well educated, they were more aware of risk taking [36]. Risk taking is likely to also reflect a lack of understanding about fertility. Hammarberg and colleagues [37] recently showed that in a representative sample of Australian women there were considerable knowledge gaps about factors that influence fertility.

Moreover another type of risk-taking was identified in this study. Nearly a fifth of the women were affected by alcohol at the time of conception of the unwanted pregnancy. The effects of alcohol may be responsible for reduced risk awareness and omission of contraceptive use at the time of sexual activity. Research on alcohol and sexual risk-taking has been reported previously although not in relation to TOP [38–40]. A meta-analysis of alcohol and condom use in developed countries found that alcohol and condom use varied depending on the type of sexual encounter [39]. Alcohol use at the first sexual encounter for adolescents was associated with decreased condom use, but not in adults [39]. However, some studies found that in relation to first intercourse with a new partner the more alcohol consumed the less likely condoms were used [39]. Other studies however found that alcohol consumption and condom use by women were associated with whether the relationship was with a primary sexual partner (were more likely to use a condom), or a nonprimary sexual partner (less likely to use a condom) [40]. The study, however, did not present information on age groups specifically although it did mention that condom use was more likely in younger participants (mean age: 24.66 both sexes included) than older participants (mean age: 29.84 both sexes included) [40]. These studies were predominantly on younger aged women. Further research into the use of alcohol which results in unwanted pregnancies in women aged over 30 years could provide further insight into women's experiences. This in turn could have implications for TOP service delivery and SRH education.

## 5. Limitations of the Study

There were a number of limitations to this study. Firstly the response rate was low. Whilst every effort was made to increase the response rate including regular contact by the researcher with the clinics collecting the data, the response rate remained low. There are several possible reasons why women did not respond to the questionnaire. The sensitive nature of TOP may have discouraged their involvement or they may have been deterred by the presence of a partner. Possible reasons are numerous however and impossible to identify to inform further recruitment. There may also have been issues in the distribution of questionnaires by clinic staff. Similar limitations in data collection were in a similar New Zealand study, which distributed questionnaires in nine TOP clinics and which also had a low response rate (36.8%) [41]. The low response rate weakens the study findings so that generalisations cannot be made.

The strengths of this study however are that the results are able to provide a greater understanding of the issues in fertility management faced by women over the age of 30 years who had an unplanned pregnancy. As there is minimal research regarding TOP in women in this age group, these research findings contribute to the body of knowledge in this little known area of health care.

## 6. Conclusion 

This research has provided insight into many issues women aged over 30 years face regarding management of their fertility prior to a TOP. This information has implications for service delivery for clinicians, policy makers, funding bodies, researchers. The overall aim is to reduce the number of unwanted pregnancies and hence the TOP rate. This paper has shown that this can only be achieved by taking a multifocal approach based on the best available evidence. To this end it is recommended that clinicians consider the fertility management needs of women over the age of 30 years and tailor sexual health education programs to include specific issues which impact on this age group. This could have a specific focus on relationship issues particularly around contraception decision making. Additionally, policy makers need to consider the different needs of this age group of women and ensure policies adequately address their specific needs and the issues which impact women's understanding of their fertility risks. Furthermore, adequate funding is required so that programs and policies can be implemented and evaluated. Finally, further research, particularly qualitative, is required to examine in more detail the issues this age group of women experience so that greater depth to understanding is available to increase the body of knowledge in this sensitive area of healthcare.

## Figures and Tables

**Table 1:** Participant demographics.

	Frequency	Valid percent
Cultural background		
Valid 90		
Australian	64	70.5
Non-Australian	26	29.5
How long in Australia		
Valid 90		
Born in Australia	64	70.9
20 to 50 years	10	11.5
10 to 19 years	7	10.0
2 to 9 years	6	5.5
1 year or less	3	2.2
Main language spoken at home		
Valid 84		
English	80	92.0
Non-English	4	8.0
Aboriginality		
Valid 90		
Aboriginal/Torres Strait Islander/both	4	4.6
Non-Aboriginal/Torres Strait	86	95.4
Islander/both		
Employment		
Valid 99		
Employed full time	32	32.2
Employed part time	26	26.6
Not in the labour force—home duties	17	17.6
Not in the labour force—on a pension,	10	9.9
government support, or carer benefit		
Employed but away from work at present	9	8.6
Unemployed looking for full-time work	3	3.1
Unemployed looking for part-time work	2	2.1
Education		
Valid 100		
Did not complete secondary school	19	18.8
Completed secondary school	23	23.2
Certificate	16	16.2
Advanced diploma, diploma	12	12.3
Bachelor degree	19	18.9
Graduate diploma, graduate certificate	3	2.6
Postgraduate degree	8	8.0
Marital status		
Valid 94		
Not married	30	32.0
Married in a registered marriage	26	27.4
Married in a de facto relationship	22	23.6
Not in a relationship	16	17.0
Partner relationship		
Valid 90		
Stable	50	55.9
New	12	13.2
Uncertain	16	17.7
No future	8	9.0
About to break up	4	4.3
Healthcare card/pension eligibility		
Valid 97		
No	52	58.0
Yes	38	42.0
Prefer not to answer	7	6.9
Age groups		
Valid 98		
30–34	44	44.9
35 plus	54	55.1
Residency		
Valid 92		
Metropolitan	75	81.8
Rural	17	18.2

**Table 2:** Obstetric history.

	Frequency	Valid percent
Number of children		
Valid 99		
This is my first pregnancy	12	12.6
Nil	11	11.7
One	19	19.5
Two	32	32.9
Three	12	12.7
Four	6	6.0
Five	2	2.4
Six	2	1.8
Seven	0	0.4
Prefer not to answer	3	3.0
Miscarriages		
Valid 99		
Nil	55	58.0
One	27	28.3
Two	11	11.6
Three or more	2	2.1
Prefer not to answer	4	4.0
Number of ectopic pregnancies		
Valid 100		
Nil	89	92.5
One	6	5.9
Two	0	0.0
Three or more	2	1.6
Prefer not to answer	3	3.0
Number of previous termination of pregnancies		
Valid 97		
Nil	45	47.9
One	22	23.2
Two	13	14.1
Three	10	10.9
Four	0	0.0
Five	2	2.5
Six	0	0.0
Seven	0	0.0
Eight	1	1.4
Prefer not to answer	4	3.8

**Table 3:** Women's fertility management experiences.

Question number		*N*	Cases (%)
	Contraception		
1	I took precautions but the contraceptive did not work (any type including ECP)	43	42.9
2	I was afraid of side effects of certain contraceptives	32	32.0
3	I was in the process of beginning a new contraceptive method	31	31.0
4	I thought it was during the safe period	31	30.8
5	I thought it could not happen to me	30	29.5
6	I just never got around to getting contraception	21	21.2
7	I had planned not to have any more intercourse for a while	19	19.3
8	There is no contraception that suits me	9	8.6
9	I felt a contraceptive would interfere with the natural expression of love	7	6.8
10	I did not like deliberately planning for the possibility of intercourse	3	3.1
11	My usual contraceptive method was not available	2	2.0
12	I felt it was wrong and against my beliefs to use contraceptives	2	1.9
13	I could not afford to buy contraception	1	0.8
14	I did not think any contraceptive really worked	1	0.8

	Pregnancy—risk-taking		
15	I put the possibility of pregnancy out of my mind	36	36.3
16	I got carried away before I could think about contraception	21	21.4
17	I did not think I would get pregnant because I have often had intercourse without protection	21	21.0
18	My judgment was affected by alcohol	19	18.6
19	I knew I might get pregnant but that uncertainty did not concern me very much	12	11.8
20	I felt that having intercourse on that occasion was worth the chance of pregnancy	12	11.5
21	I did not think pregnancy was likely because I had intercourse so infrequently	11	11.0
22	My judgment was affected by a drug (marijuana, speed, ecstasy, etc.)	4	4.4
23	I sort of liked putting myself in a risky situation	3	3.1

	Fertility		
24	I did not think I was fertile	20	20.3
25	At the time I wanted to get pregnant	3	3.1
26	I wanted to prove I was fertile	0	0.3

	Attitudes		
27	I realised that if I did get pregnant I could get an abortion	9	9.0

	Partner/relationship		
28	He was supposed to withdraw but he did not	13	13.2
29	He wanted to have a baby even though I did not	9	8.6
30	We could not agree what kind of contraceptive method to use	8	7.8
31	He did not want me to use contraception	7	7.4
32	I loved him and nothing else mattered	7	6.7
33	He would not/will not use contraception (condoms, withdrawal, and vasectomy)	6	5.7
34	I was not able to take precautions because he was in such a hurry	3	3.1
35	I thought that if I got pregnant it would improve our relationship	3	2.9
36	I thought that if I got pregnant we would stay together	3	2.6
37	I was not confident to talk to him about contraception	2	1.8
38	He assured me he was sterile or that he could not make me pregnant	2	1.8
39	I thought that using contraception would make it harder to say no in future	1	1.0
40	I thought that using a contraceptive would change how he felt about me	0	0.0
41	Sex was unwanted	0	0.0

	Doctor issues		
42	I could not get to see a doctor for contraception	5	5.1
43	The doctor gave me the wrong information	3	2.7
44	The doctor refused to give me contraception	0	0.3

## References

[1] Lattimore R, Pobke C Recent trends in Australian fertility. http://www.pc.gov.au/__data/assets/pdf_file/0003/82371/recent-fertility-trends.pdf.

[2] Miranti R, McNamara J, Tanton R, Yap M (2009). A narrowing gap? Trends in the childlessness of professional women in Australia 1986–2006. *Journal of Population Research*.

[3] Australian Bureau of Statistics Total Fertility Rate: 1367.0—State and Territory Statistical Indicators, 2011. http://www.abs.gov.au/ausstats/abs@.nsf/Lookup/by+Subject/1367.0~2011~Main+Features~Total+Fertility+Rate~5.2.

[4] Loxton D, Lucke J (2009). *Reproductive health: findings from the Australian Longitudinal Study on Women's Health*.

[5] Lutz W, Leridon H, Aitken RJ, Von Eyben FE (2006). Fertility rates and future population trends: will Europe’s birth rate recover or continue to decline?. *International Journal of Andrology*.

[6] Mills M, Rindfuss RR, McDonald P, te Velde E (2011). Why do people postpone parenthood? Reasons and social policy incentives. *Human Reproduction Update*.

[7] Abigail W, Power C (2008). A systematic review of trend studies of women seeking termination of pregnancy. *Journal of Clinical Nursing*.

[8] Doskoch P (2012). Global abortion rate stabilizes, but unsafe procedures remain the norm in developing countries. *International Perspectives on Sexual & Reproductive Health*.

[9] Rice K (2005). *Abortion Issues Paper*.

[10] Pratt A, Biggs A, Buckmaster L How many abortions are there in Australia? A discussion of abortion statistics, their limitations, and options for improved statistical collection.

[11] Chan A, Sage LC (2005). Estimating Australia’s abortion rates 1985–2003. *Medical Journal of Australia*.

[12] Calcutt C (2007). Abortion services in Australia. *O & G Magazine*.

[13] Chan A, Scott J, Nguyen A-M, Sage L (2009). *Pregnancy Outcomes in South Australia 2008*.

[14] Abigail WF, Power C, Belan I (2010). Termination of pregnancy and the over 30s: what are trends in contraception use 1996–2006?. *Australian Journal of Primary Health*.

[15] Abigail W The known and unknown of older women's experiences of fertility management/control prior to a termination of pregnancy: a systematic review.

[16] ABS 3101. 0-Australian demographic statistics, June 2008. http://www.abs.gov.au/ausstats/abs@.nsf/mf/3101.0.

[17] Australian Bureau of Statistics (2007). *Population by Age and Sex, Regions of Australia, 2007*.

[18] ABS (2006). *2006 Census of Population and Housing: South Australia Occupation by Sex, Health Occupations*.

[19] Australian Bureau of Statistics Australian Bureau of Statistics. http://www.abs.gov.au/websitedbs/d3310114.nsf/home/Census+data.

[20] Ivankova NV, Creswell JW, Stick SL (2006). Using mixed-methods sequential explanatory design: from theory to practice. *Field Methods*.

[21] ABS Survey or census: what's the difference. http://www.abs.gov.au/websitedbs/a3121120.nsf/4a256353001af3ed4b2562bb00121564/8dac06eb98d00563ca2576710002e748!OpenDocument.

[22] Tucker G, Sage L (2008). *Pregnancy Outcomes Statistics*.

[23] Raosoft Inc (2004). *Sample Size Calculator*.

[24] Miller WB (1975). Psychological antecedents to conception among abortion seekers. *Western Journal of Medicine*.

[25] Wood MJ, Ross-Kerr JC (2006). *Basic Steps in Planning Nursing Research: From Question to Proposal*.

[26] Gray E, McDonald P Contraceptive practice and the reproductive life course.

[27] Gray E, McDonald P (2010). Using a reproductive life course approach to understand contraceptive method use in Australia. *Journal of Biosocial Science*.

[28] Sedgh G, Bankole A, Singh S, Eilers M (2013). Legal abortion levels and trends by woman's age at termination legal abortion levels and trends by woman's age at termination. *Perspectives on Sexual & Reproductive Health*.

[29] Dominguez-Folgueras M, Castro-Martin T (2013). Cohabitation in Spain: no longer a marginal path to family formation. *Journal of Marriage and Family*.

[30] Cooke A, Mills TA, Lavender T (2010). ’Informed and uninformed decision making’-Women’s reasoning, experiences and perceptions with regard to advanced maternal age and delayed childbearing: a meta-synthesis. *International Journal of Nursing Studies*.

[31] Kero A, Högberg U, Lalos A (2001). Contraceptive risk-taking in women and men facing legal abortion. *European Journal of Contraception and Reproductive Health Care*.

[32] Törnbom M, Möller A (1999). Repeat abortion: a qualitative study. *Journal of Psychosomatic Obstetrics and Gynaecology*.

[33] Helström L, Odlind V, Zätterström C (2003). Abortion rate and contraceptive practices in immigrant and native women in Sweden. *Scandinavian Journal of Public Health*.

[34] Gaydos LMD, Hogue CJR, Kramer MR (2006). Riskier than we thought: revised estimates of noncontracepting women risking unintended pregnancy. *Public Health Reports*.

[35] Kumar U, Baraitser P, Morton S, Massil H (2004). Peri-abortion contraception: a qualitative study of users’ experiences. *Journal of Family Planning and Reproductive Health Care*.

[36] Moreau C, Bouyer J, Goulard H, Bajos N (2005). The remaining barriers to the use of emergency contraception: perception of pregnancy risk by women undergoing induced abortions. *Contraception*.

[37] Hammarberg K, Setter T, Norman RJ, Holden CA, Michelmore J, Johnson L (2013). Knowledge about factors that influence fertility among Australians of reproductive age: a population-based survey. *Fertility and Sterility*.

[38] Parks KA, Hsieh Y-P, Collins RL, Levonyan-Radloff K, King LP (2009). Predictors of risky sexual behavior with new and regular partners in a sample of women bar drinkers. *Journal of Studies on Alcohol and Drugs*.

[39] Leigh BC (2002). Alcohol and condom use: a meta-analysis of event-level studies. *Sexually Transmitted Diseases*.

[40] Scott-Sheldon LAJ, Carey MP, Vanable PA, Senn TE, Coury-Doniger P, Urban MA (2009). Alcohol consumption, drug use, and condom use among STD clinic patients. *Journal of Studies on Alcohol and Drugs*.

[41] Silva M, McNeill R, Ashton T (2010). Ladies in waiting: the timeliness of first trimester services in New Zealand. *Reproductive Health*.

